# TGF-β Signaling in Cellular Senescence and Aging-Related Pathology

**DOI:** 10.3390/ijms20205002

**Published:** 2019-10-10

**Authors:** Kana Tominaga, Hiroshi I. Suzuki

**Affiliations:** 1 Department of Biology and Paul F. Glenn Center for Biology of Aging Research, Massachusetts Institute of Technology, Cambridge, MA 02139, USA; kanat@mit.edu; 2 David H. Koch Institute for Integrative Cancer Research, Massachusetts Institute of Technology, Cambridge, MA 02139, USA

**Keywords:** transforming growth factor-β (TGF-β), cellular senescence, stem cell, aging

## Abstract

Aging is broadly defined as the functional decline that occurs in all body systems. The accumulation of senescent cells is considered a hallmark of aging and thought to contribute to the aging pathologies. Transforming growth factor-β (TGF-β) is a pleiotropic cytokine that regulates a myriad of cellular processes and has important roles in embryonic development, physiological tissue homeostasis, and various pathological conditions. TGF-β exerts potent growth inhibitory activities in various cell types, and multiple growth regulatory mechanisms have reportedly been linked to the phenotypes of cellular senescence and stem cell aging in previous studies. In addition, accumulated evidence has indicated a multifaceted association between TGF-β signaling and aging-associated disorders, including Alzheimer’s disease, muscle atrophy, and obesity. The findings regarding these diseases suggest that the impairment of TGF-β signaling in certain cell types and the upregulation of TGF-β ligands contribute to cell degeneration, tissue fibrosis, inflammation, decreased regeneration capacity, and metabolic malfunction. While the biological roles of TGF-β depend highly on cell types and cellular contexts, aging-associated changes are an important additional context which warrants further investigation to better understand the involvement in various diseases and develop therapeutic options. The present review summarizes the relationships between TGF-β signaling and cellular senescence, stem cell aging, and aging-related diseases.

## 1. Introduction

Aging is broadly defined as the time-dependent process of the gradual decline in the physiological function, adaptability, and endurance of tissues and organs over a lifetime [1,2]. The aging process varies individually and is affected by genetic background and calorie consumption [3]. At the cellular level, the loss of cell homeostasis is associated with the aging process. Approximately 60 years ago, aging was suggested to be caused by the accumulation of free radicals, OH, and/or HO_2_ in the cells [4]. This type of cellular damage is attributable to increased oxidative stress and/or impaired DNA damage repair mechanisms, further enhancing the accumulation of DNA damage, which is a hallmark of both aging and carcinogenesis. However, aging may be driven by the alteration of various other intracellular macromolecules including proteins and lipids. To preserve the functionality of numerous proteins in a wide range of environmental and metabolic conditions, all protein species need to efficiently fold and assemble during synthesis. In recent studies, the capacity to maintain protein homeostasis has been shown to possibly decline below a critical threshold of activity required to manage typical oxidative stress levels in the normal state, contributing to age-related diseases. Thus, aging can also be considered a phenomenon caused by the malfunction of protein homeostasis (proteostasis) or balance between proteins [5,6,7].

The transforming growth factor-β (TGF-β) is a superfamily of evolutionarily conserved cytokines that control pleiotropic cellular functions [8]. In mammals, this family has 33 members including TGF-βs, activins, bone morphogenetic proteins (BMPs), and growth differentiation factors (GDFs). Humans have three subtypes of TGF-β: TGF-β1, -β2, and -β3. TGF-β signaling contributes to diverse cell processes such as cell proliferation, migration, differentiation, and apoptosis in various cell types; notably, TGF-β is involved in multiple aspects of cancer biology and thus provides important options as a valid therapeutic target [9].

Though our understating of the aging process remains limited, and the context-dependent and multifunctional activities of TGF-β frequently complicate the interpretation of its in vitro and in vivo effects, recent reports have directly and indirectly linked TGF-β signaling to aging-related processes. TGF-β exerts diverse functions by modulating the expression of downstream target genes via transcriptional and post-transcriptional mechanisms as well as protein modulation in a context-dependent manner. Importantly, the downstream targets of TGF-β signaling include many regulators involved in multiple aspects of aging processes, such as cell proliferation, cell cycle regulation, the production of reactive oxygen species (ROS), DNA damage repair, telomere regulation, unfolded protein response (UPR), and autophagy. Due to a large overlap between the two pathways, TGF-β signaling exhibits multifaceted crosstalk with aging processes. At the cellular level, TGF-β signaling has been shown to play an important role in cellular senescence and stem cell aging. Furthermore, the alteration of TGF-β signaling pathways has been frequently observed in various age-related diseases, including cardiovascular disease, Alzheimer’s disease (AD), osteoarthritis, and obesity.

In the present review, we summarize the relationships between TGF-β signaling and aging, cellular senescence, and aging-related diseases with an emphasis on the findings obtained in human and rodent studies.

## 2. Cellular Senescence and Aging

The accumulation of senescent cells is considered a hallmark of aging and thought to contribute to aging pathologies. Once several stresses including ROS and DNA replication accumulate in replication-competent and proliferative cells, cells undergo near permanent cell cycle arrest while remaining viable and metabolically active [1,2,10]. Hayflick observed cellular senescence as the irreversible loss of replicative capacity in primary somatic cell culture [11]. Mammalian senescent cells are characterized by large and flat shapes and increased endogenous senescence-associated β-galactosidase (SA-β-gal) activity. In human fibroblasts, morphological changes associated with senescence are regulated by the UPR [12]. During senescence, irreversible growth arrest is mediated by the activation of two major tumor suppressor pathways regulated by p16^Ink4a^/retinoblastoma (Rb) and p53/p21. Cellular senescence is considered a barrier to tumorigenesis, and the mutation of these tumor suppressor genes can transform senescence cells into cancer cells.

Another important feature of cellular senescence is the senescence-associated secretory phenotype (SASP), in which senescent cells secrete multiple cytokines, chemokines, extracellular matrix (ECM) proteins, and ECM regulators during the process of senescence following cellular damage [1,2,13]. The SASP can contribute to tumor suppression by reinforcing the induction of a senescent phenotype in an autocrine/paracrine fashion and recruiting immune cells that remove damaged or oncogene-expressing cells from organisms [2]. Conversely, the SASP is thought to also have pro-tumorigenic effects by promoting inflammation and ECM remodeling in the tumor microenvironment, indicating the complex roles of the SASP in tumor progression [13,14,15]. The SASP appears to participate in age-related pathological processes [15]. The depletion of senescent cells using genetic engineering and senolytic drug administration has been shown to delay aging-related disorders and alleviate physical dysfunction in mice [16,17].

## 3. Regulation of Cellular Proliferation and Senescence by TGF-β Signaling

The intracellular signaling of TGF-β family ligands is mediated by specific cell-surface serine-threonine receptors and intracellular mediators known as Smad proteins. Ligand binding induces the assembly of a hetero-tetrameric complex of two type I and two type II receptors. Characteristic combinations of seven type I receptors (activin receptor-like kinase 1-7, ALK1-7) and five type II receptors (TβRII, ActRIIA, ActRIIB, BMPRII, and AMHRII) correspond to signal transduction by individual members of the TGF-β family [18,19]. The three TGF-β ligands mainly bind to the TGF-β type II receptors (TβRII) and type I receptors (TβRI, also known as ALK5). Activins act through the type II receptors ActRII or ActRIIB and type I receptors ALK4 or ALK7. BMP signaling is mainly mediated by the type II receptors ActRII, ActRIIB, or BMPRII and type I receptors ALK2, ALK3, or ALK6. In contrast to TβRII, other type II receptors ActRIIA, ActRIIB, and BMPRII interact with a large group of overlapping ligands at overlapping epitopes, contributing to cross-inhibition between different TGF-β family ligands [20]. After TGF-β ligands bind to cell-surface type II and type I receptors, receptor complexes are activated via serine/threonine protein kinase, and type I receptors phosphorylate the receptor-regulated class of Smads (R-Smads) [8]. Type I receptors for TGF-βs and activins induce the phosphorylation of Smad2 and Smad3 (activin/TGF-β-specific R-Smads), and BMP type I receptors induce the phosphorylation of Smad1, Smad5, and Smad8 (BMP-specific R-Smads). The activated R-Smads form complexes with a common-partner Smad (co-Smad), Smad4. The R-Smad/co-Smad complexes then translocate to nuclei and regulate the expression of various target genes, mainly as transcriptional regulators.

TGF-β has been shown to have dual functions in cancer biology: An early tumor suppressor and a late tumor promoter [9]. The former has been attributed to the potent growth inhibitory and pro-apoptotic activities of TGF-β in various cell types, although TGF-β can also promote cell proliferation in several cell types. In the later stage, TGF-β is thought to stimulate tissue fibrosis and ECM deposition, enhance cell migration and metastasis, and perturb immune and inflammatory reactions in the tumor microenvironment.

The cytostatic effects of TGF-β are mediated by inducing the cyclin-dependent kinase inhibitors p15^Ink4b^, p21, and p27, and by suppressing several proliferation factors including c-Myc [21,22]. This suggests a senescence promoting role of TGF-β under normal conditions and also coincides with the tumor suppressing role of cell senescence. TGF-β has been shown to induce or accelerate senescence and senescence-associated features in various cell types, including fibroblasts, bronchial epithelial cells, and cancer cells [23,24,25]. In addition, the TGF-β-mediated accumulation of senescent cells has been suggested in idiopathic pulmonary fibrosis (IPF) [24].

**Figure 1:**
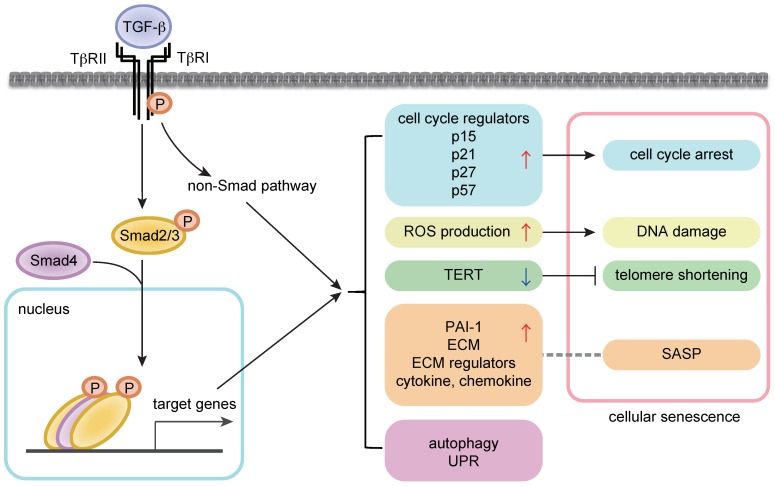
TGF-β (transforming growth factor-β) signaling in cellular senescence. (**Left**) Intracellular signal transduction cascades by TGF-β. (**Right**) The link between TGF-β signaling and senescence modulators. The arrows and T bar indicate enhancement and suppression, respectively. A dot line indicates the relationship between TGF-β downstream targets and SASP.

In addition to the cytostatic mechanisms, the senescence-promoting role of TGF-β might be explained by the effects on other modulators of senescent phenotypes [21,22] (Figure 1). TGF-β reportedly induces ROS production in the mitochondria in several cell types including hepatocytes and lung epithelial cells [26,27]. In addition, TGF-β suppresses telomerase activities by downregulating the expression of telomerase reverse transcriptase (TERT) in various cell types including rat fibroblasts and human breast cancer cells [28,29]. TGF-β regulates various non-coding RNAs including microRNAs [30,31]. A recent report showed that TGF-β signaling induced the reduction of H4K20me3 abundance, which compromised DNA damage repair and promoted senescence, by upregulating miR-29a/c and downregulating its target Suv4-20h in fibroblasts [28]. This pathway contributed to cardiac aging in vivo, and the inhibition of TGF-β signaling restored H4K20me3 and improved cardiac function in older mice [32].

TGF-β induces the expression of inhibitory Smads (I-Smads), Smad6 and Smad7, thus incorporating negative feedback mechanisms. This type of regulation may also be utilized for modulating senescence. In bronchial epithelial cells, TGF-β causes senescence by inducing p21 as well as the expression of sirtuin 6 (SIRT6), a class III histone deacetylase, which counteracts senescence [24]. In IPF lung samples, an increase in markers of senescence and SIRT6 expression was observed in bronchial epithelial cells [24]. Though the induction of SIRT6 is insufficient to prevent TGF-β-induced senescence, the overexpression of SIRT6 has been shown to inhibit senescent phenotypes via p21 degradation. In addition, although the impact regarding senescence regulation has not been well studied, we and others have reported that TGF-β modulates protein homeostasis by regulating UPR and autophagy pathways [33,34,35].

The SASP yields the production and secretion of various signaling molecules, importantly including TGF-β. Thus, TGF-β is secreted as one of the SASP factors and can induce and maintain senescent phenotype and age-related pathological conditions in an autocrine/paracrine manner. In a recent study, integrin β3 (ITGB3), regulated by the polycomb protein CBX7, was upregulated during senescence, promoted senescence by activating TGF-β signaling in an autocrine/paracrine manner, and reinforced the SASP in human fibroblasts [36]. The study also indicated a positive correlation between the expression levels of ITGB3 and several TGF-β signaling components such as TβRI, TβRII, Smad3, and Smad4, and aging [36]. Accordingly, several studies have shown that the plasma level of active TGF-β1 increased significantly in elderly humans compared with younger subjects [37,38]. The relationship between TGF-β signaling and longevity and age-related pathological conditions is discussed later in this review. In addition, plasminogen activator inhibitor-1 (PAI-1), a well-known TGF-β-induced target, is an important mediator of senescence and age-related pathology [39].

## 4. Regulation of Stem Cell Aging by TGF-β Signaling

In addition to cellular senescence, aging processes can be viewed at the cellular level as a functional decline of stem cells in somatic tissues, which govern tissue regeneration to maintain physiological homeostasis [40,41,42]. Adult stem cells exhibit a capacity for self-renewal and differentiation [40,41,42]. In somatic cells, telomere length becomes shorter per cell division and is associated with progressive aging. Tissue stem cells as well as differentiated cells are strongly influenced by the accumulation of DNA damage such as the reduction of telomere length and genomic instability, finally leading to reduced regeneration capacity [43,44,45]. Senescence is a contradiction in terms with respect to stem cells because a permanently arrested stem cell is no longer a stem cell, and stem cell aging or quiescence are the preferred terms in stem cell biology, while senescence is often used to describe the phenotypes of mesenchymal stem cells (MSCs). TGF-β has been shown to exert pleiotropic effects on the regulation of various tissue-specific stem cells, such as MSCs, hematopoietic stem cells (HSCs), neural stem cells, and hair follicle stem cells [46]. In this section, the role of TGF-β signaling in MSCs and HSCs is summarized.

### 4.1. Mesenchymal Stem Cells

MSCs are widely defined as a plastic-adherent cell population derived from bone marrow or other tissues including adipose tissue that expands and induces the formation of bone, cartilage, muscle, and other skeletal tissues in culture [47]. Though their characterization largely depends on in vitro culture studies, MSCs are thought to have important roles in supporting blood vessels, maintaining bone homeostasis, and modulating immune function through their capacity for differentiation as well as the secretion of various cytokines and chemokines including TGF-β. MSC-derived TGF-β has been proposed to modulate immune function and protect cancer cells in the tumor microenvironment [48]. In several studies, aged MSCs showed a decreased proliferation ability and an altered differentiation potential, and they were accompanied by typical senescent features, including the accumulation of ROS, p16, and p21, and increased SA-β-gal activity [49,50,51]. While MSC differentiation favors osteoblastogenesis in young adults, the differentiation is balanced toward adipogenesis in elderly adults [52,53].

Reports on the effects of TGF-β on proliferation and senescence of MSCs have varied in the literature. The senescence-promoting activities of TGF-β in several MSC types, including bone marrow-derived MSCs and endometrial MSCs, have been previously described [54,55]. In bone marrow-derived MSCs, TGF-β reportedly increases the expression levels of aging markers, p16^Ink4a^ and 4-Hydroxynonenal (4-HNE) subunits, SA-β-gal activity, and the production of mitochondrial ROS in a dose-dependent manner [54]. However, other studies have suggested that TGF-β promotes the proliferation of MSCs or does not induce senescence [56,57,58]. These contradictory observations might be partly explained by the heterogeneity of MSCs, as human MSCs are known to contain at least three types which possess osteo-chondro-adipogenic, osteo-chondrogenic, and only osteogenic differentiation capacities [59]. In addition, it is known that fibroblasts derived from different tissues display distinct gene expression patterns and express different sets of transcription factors [60,61,62]. Such heterogeneity may be also the case for MSCs in different tissues and may be associated with differential responses to TGF-β. Furthermore, potent effects of TGF-β on MSC differentiation may confound the interpretation of growth effects associated with TGF-β signaling. TGF-β promotes chondroblast differentiation at early stages and inhibits osteoblast maturation at late stages [63]. TGF-β also inhibits adipocyte differentiation and stimulates myofibroblastic differentiation. Collectively, the precise effects of TGF-β on the senescent phenotype of MSCs remain unclear and require a better characterization of MSC identity. From this standpoint, importantly, recent reports using human bone-derived CD271 and SSEA-4 double positive MSCs (DPMSCs), which support HSCs and lack adipogenic differentiation potential, have demonstrated that TGF-β could accelerate the senescence of DPMSCs and tends to reduce their hematopoiesis-supporting ability [64,65]. Furthermore, one of these studies found that old DPMSCs showed higher TGF-β2 expression levels than young DPMSCs, indicating the senescent phenotypes and reduced hematopoiesis-supporting ability of old DPMSCs may involve the activation of TGF-β signaling [65].

### 4.2. Hematopoietic Stem Cells

HSCs are the source of blood cells existing in the bone marrow and can differentiate into erythrocytes, leukocytes, and platelets. The HSC population is characterized by the c-Kit^+^/Lin^−^/Sca-1^+^ (KLS) phenotype in mice and is largely grouped into long-term and short-term HSCs. Based on multiple in vitro and in vivo studies, TGF-β is considered a negative regulator of hematopoiesis and a candidate cytokine that induces HSC quiescence [66]. Mechanistically, in contrast to the growth inhibitory mechanisms of TGF-β in adherent cells, TGF-β can induce the cell cycle arrest of hematopoietic cells independent of p21 and p27. In human CD34^+^ cells and mouse CD34^−^ KLS cells, TGF-β upregulated p57 to induce cell cycle arrest [67,68]. A high level of p57 expression and Smad2/3 phosphorylation has been observed in CD34^−^ KLS cells relative to cycling CD34^+^ KLS fraction, indicating the TGF-β signaling is highly active in dormant HSCs [68]. In addition, the results from a recent study using an in vitro single cell culture and an in vivo repopulating assay showed that TGF-β1 decelerated the cell cycle progression of HSCs and consequently attenuated their self-renewal capacity [69].

These findings have largely been supported by several knockout mouse models of TGF-β signaling components. TGF-β1-null mice develop a lethal inflammatory disease, reflecting the immunosuppressive activity; however, bone marrow cells from TGF-β1-deficient neonates have reportedly showed an impaired reconstitutive activity and a decreased homing capacity in transplantation settings [70]. In another report, the conditional depletion of TβRII led to increased HSC cycling and a reduced reconstitutive capacity [71]. Furthermore, the inhibition of TGF-β signaling with TGF-β-neutralizing antibodies and Smad4 overexpression enhanced and reduced hematopoietic recovery following stress, respectively [72,73]. Conversely, the depletion of TβRI does not influence HSC behavior [74,75] possibly due to low TβRI expression levels in HSCs; this lack of influence may indicate alternative signaling cascades in this cell type or the importance of the aging-associated alteration of TβRI expression, as discussed later.

Aging in the hematopoietic system manifests several features: (1) Myeloid-biased HSCs become abundant with advancing age, and lymphoid-biased HSCs decrease; (2) CD41 expression increases with age to mark the majority of long-term HSCs in mice [76,77]. The ranscriptome analysis of HSCs obtained from young (four months) and older (24 months) mice identified the widespread alteration of TGF-β-regulated genes, exemplified by the downregulation of Nr4a1, Cebpa, Jun, and Junb, as one of the remarkable transcriptome changes in old HSCs, suggesting reduced TGF-β signaling [78]. However, this finding may not simply indicate the downregulation of TGF-β signaling but the association with a more complicated context-dependent alteration of TGF-β signaling. In a recent report, transcriptional intermediary factor 1γ (Tif1γ), another transcriptional partner of Smad2/3, was downregulated in old HSCs, and the depletion of Tif1γ in HSCs accelerated the aging phenotype in mice [79]. Notably, Tif1γ regulated TβRI expression levels, and Tif1γ-depleted and old HSCs showed higher TβRI expression levels, Smad2/3 phosphorylation, and sensitivity to TGF-β-mediated cytostasis than young HSCs [79]. Thus, the alteration of a balance between Smad2/3-Smad4 complex, Smad2/3-Tif1γ, and other Smad2/3-associated protein complexes may modulate the sensitivity to TGF-β and further redirect transcriptional responses to TGF-β in aging processes. In that respect, several previous complicated observations from in vitro studies, such as a biphasic response of HSCs to TGF-β2 and differential responses of myeloid-biased HSCs and lymphoid-biased HSCs to TGF-β, may clarify the understanding of the relationship between TGF-β and an aged hematopoietic system [80,81].

## 5. TGF-β Signaling in Longevity and Aging-Related Pathology

As described earlier, aging and fibroblast senescence correlate with increased TGF-β signaling. At the organism level, aging is associated with altered levels of several circulating cytokines, such as increased levels of plasma IL-6, IL-1 receptor antagonist, and active TGF-β [37,82]. In a study with Italian centenarians, an association between TGF-β1 polymorphism (NM_000660.7(TGFB1):c.74G>C (p.Arg25Pro)) and human longevity was suggested; the G allele, which correlated with increased TGF-β production, was more frequently observed in centenarians than in the young control group, while active TGF-β1 levels in plasma did not correlate with TGF-β genotype in the study [38,83].

**Figure 2:**
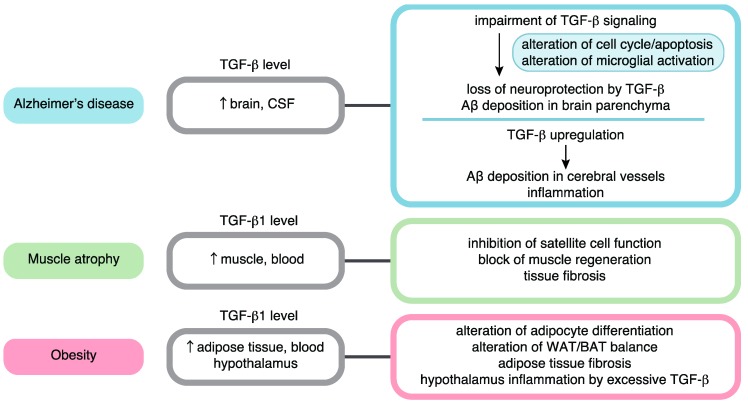
TGF-β signaling in aging-related disease pathology. Roles in Alzheimer’s disease, muscle atrophy, and obesity are summarized.

More specifically, TGF-β signaling has been suggested to contribute to the mechanisms of various age-related diseases. In the latter half of this review, we summarize the role of TGF-β signaling in age-related conditions by focusing on AD, muscle atrophy, and obesity (Figure 2). A complex interplay among TGF-β signaling, aging, and cancer has been reviewed and discussed by others [21]. In addition, the relationships between TGF-β signaling and cardiovascular disease, lung fibrosis, and osteoarthritis have been discussed in other reviews [84,85,86,87].

## 6. Roles of TGF-β Signaling in Alzheimer’s Disease

TGF-β has been characterized as an injury-responsive factor and subsequently as a disease modulator in the central nervous system (CNS) based on the observation that among three TGF-β ligands, TGF-β1 increases acutely after brain injury, including trauma, ischemia, and encephalitis, and chronically in many neurodegenerative diseases, including AD, Parkinson’s disease, and amyotrophic lateral sclerosis (ALS) [88,89,90]. TGF-β1 also increases with aging [91]. In this context, TGF-β signaling elicits complicated effects (e.g., beneficial or detrimental) including neuroprotection, gliosis, hydrocephalus, and vascular fibrosis, depending on the cellular, spatial, and temporal context [88,89,90].

Alzheimer’s disease is one of the most common chronic CNS disorders in older people and is pathologically characterized by the accumulation of β-amyloid peptide (Aβ) in the brain parenchyma and blood vessel walls, neurofibrillary tangles, and neuronal loss [92]. In AD, TGF-β levels increase in the brain tissue and cerebrospinal fluid (CSF) [93,94,95,96]. Though reports on the plasma TGF-β levels have varied in the literature [96,97,98,99,100,101,102], the elevation of TGF-β levels in both CSF and plasma was suggested in a meta-analysis study [103]. Conversely, the impairment of TGF-β signaling in neurons has been suggested as a common feature of AD. The cytoplasmic retention of phosphorylated Smad2/3 and its colocalization with amyloid plaques and neurofibrillary tangles has been observed in the hippocampal neurons in the brains of AD patients, indicating the inhibitory effects of neurofibrillary tangles on TGF-β signaling [104,105]. Reduced neuronal TβRII expression has also been observed in the early phase of AD and has been correlated with the pathological hallmarks of AD [106]. This observation is specific to AD among several neurodegenerative diseases, including Parkinson’s disease, Pick’s disease, and frontotemporal dementia [106]. In addition, an association between TGF-β1 polymorphism (NM_000660.7(TGFB1):c.29C>T (p.Pro10Leu)) and a risk of AD was suggested in a genetic study; the C allele, which is associated with reduced serum TGF-β1 levels, was found to be overrepresented in AD patients [107]. Consistent with these findings, TGF-β1 promoted Aβ clearance in microglial cell cultures [108]. Furthermore, the inhibition of TGF-β signaling increased neuritic degeneration and Aβ production in neuroblastoma cells [106,108]. These findings collectively indicate that the impairment of neuroprotective activity of TGF-β contributes to AD. The neuroprotective roles of TGF-β have been further supported in several mouse studies. The overexpression of TGF-β1 in astrocytes and kinase-deficient TβRII in neurons has been found to attenuate and promote neurodegeneration and overall Aβ accumulation in a mouse AD model, respectively [106,108]. In a recent study, TGF-β1 overexpression restored hippocampal synaptic plasticity and memory in an AD model [109]. The TGF-β-mediated neuroprotection mechanism has not been well defined, and several mechanisms, such as the inhibition of apoptosis, synergy with neurotrophins, and microglial activation with enhanced Aβ clearance, have been postulated [90].

Conversely, in several studies, the non-neuronal effects of TGF-β signaling have appeared to modulate and deteriorate several pathological features of AD, including cerebrovascular Aβ deposition and amyloid angiopathy. Cortical TGF-β1 mRNA levels in AD patients correlated positively with the degree of cerebral amyloid angiopathy but inversely with the deposition of Aβ in plaques [108], indicating differential effects of TGF-β on Aβ deposition in cerebral vessels and brain parenchyma. TGF-β1 overexpression resulted in accelerated cerebrovascular amyloidosis and impaired cognitive function in a mouse AD model, development of AD-like vascular alterations, and reduced brain tissue perfusion [110,111,112]. In addition, another report found that the blockade of TGF-β-Smad2/3 signaling in peripheral macrophages attenuated parenchymal and cerebrovascular Aβ deposition partly through increased Aβ phagocytosis [113]. These non-neuronal roles of TGF-β signaling may be partly explained by TGF-β-modulated inflammatory responses, referred to as “neuroinflammation” [88]. Though TGF-β1 is generally considered an anti-inflammatory cytokine, a high level of TGF-β production in the brain vessels of AD patients reportedly contributed to the increased production of several pro-inflammatory cytokines, such as IL-1β and TNF-α [114]. Consistent with this, it was recently reported that TGF-β signaling in the endothelium exerted pro-inflammatory effects and promoted vascular inflammation and atherosclerosis [115].

Taken together, these results indicate that TGF-β generally has neuroprotective roles in AD, and that cell-type-dependent, spatial, and temporal contexts may differentially determine the influence of TGF-β on AD pathogenesis with a spatiotemporal balance of TGF-β production. Though Aβ deposition has been used as a marker of AD progression in many studies, a differential association between TGF-β and Aβ deposition in cerebral blood cells and brain parenchyma and the possible different functional consequences should be considered when determining the use of TGF-β modulation as AD therapy. In addition, understanding the inter-individual heterogeneity of AD, including the degrees of vascular dysfunction and the considerable differences between mouse and human brains, is important for future clinical translation [116].

## 7. Roles of TGF-β Signaling in Age-Related Muscle Atrophy

TGF-β signaling also has pleiotropic roles in muscle development, adaptation, and diseases [117,118]. The chronic elevation of TGF-β1 and/or myostatin (also known as GDF-8) has been linked with the pathology of sarcopenia and other forms of muscle atrophy associated with disuse and cachexia, frequently observed in elderly patients. Sarcopenia refers to the age-related loss of skeletal muscle mass and strength. Histologically, aging muscle exhibits increased fiber size heterogeneity, fiber type grouping, and the co-expression of multiple myosin heavy chain (MHC) isoforms, indicating the importance of sporadic denervation and repeating cycles of denervation and reinnervation in the sarcopenia mechanisms [119]. Conversely, disuse- and cachexia-associated muscle atrophy is not necessarily associated with aging, and the above-mentioned age-related histological changes have not typically been observed in experimental models of cancer cachexia, thus indicating a mechanistic difference between sarcopenia and other types of muscle atrophy, i.e., degenerative muscle atrophy—although the latter has frequently been observed with older age and may simultaneously occur with the former.

The elevation of TGF-β1 and/or myostatin in aging conditions has been described in several studies. While TGF-β1 has been well known to be upregulated in regenerating skeletal muscle after injury, TGF-β expression and Smad3 phosphorylation have also been found to increase in the skeletal muscle of older mice [120]. Increased myostatin expression has also been reported in the muscle of older people [121]. In addition, elevated serum TGF-β1 levels has been observed in both older mice and humans [122]. TGF-β1 has been suggested to contribute to muscle atrophy by blocking muscle regeneration and inducing tissue fibrosis in both human and mouse muscle tissues [117,118,122]. The effects of TGF-β signaling on muscle regeneration have mainly been investigated by researching the effects on muscle satellite cells (muscle stem cells). Satellite cells reside beneath the basal lamina surrounding muscle fibers and differentiate to myoblasts upon damage, and their activity gradually declines during aging. TGF-β1 upregulates several cyclin-dependent kinase inhibitors (p15, p16, p21, and p27), inhibits satellite cell activation, and impairs myocyte differentiation [120,122,123]. The inhibition of TGF-β signaling has consistently been shown to improve the regenerative capacity of skeletal muscle and muscle function in older animals [120,122]. The inhibition of TGF-β signaling by the ActRIIB antagonist appears to also ameliorate cachexia [124].

Unlike the contribution to muscle regeneration, the direct involvement of TGF-β signaling in sarcopenia or denervation-mediated muscle atrophy remains unclear. In a recent report, the depletion of satellite cells in adult sedentary mice did not affect sarcopenia while impairing regenerative capacity, thus indicating satellite cell-independent mechanisms in sarcopenia. Because myostatin increases in denervation-induced muscle atrophy [125], future studies may delineate the differential contribution of TGF-β signaling to denervation-induced and degenerative muscle atrophy.

Aging also induces changes in cardiac structure and function. Cardiac aging is associated with the hypertrophy and fibrosis of the heart, leading to diastolic dysfunction and heart failure [126]. TGF-β is also involved in the pathological remodeling and damage response of muscle tissue in other settings such as cardiac fibrosis and eccentric muscle contraction [84,127]. TGF-β1 expression increases during the development of cardiac hypertrophy, dilated cardiomyopathy, and aortic stenosis, as well as after myocardial infarction. The increase in local myocardial TGF-β promotes the transition of cardiac fibroblasts into myofibroblasts, leading to cardiac fibrosis and dysfunction.

## 8. Roles of TGF-β Signaling in Obesity

Obesity is a worldwide health concern and is associated with an increased risk of insulin resistance, type 2 diabetes, and cardiovascular diseases [128]. During aging, obesity is also associated with a decline in the musculoskeletal system including sarcopenia and muscle atrophy [129]. Obesity is defined as the accumulation of excess white adipose tissue (WAT) and also accompanies the reduction of brown adipose tissue (BAT) and/or inducible brown adipocytes (also called brite or beige adipocytes) in WAT [130]. WAT stores energy and is characterized by large lipid droplets, while BAT and brown adipocytes mediate energy expenditure due to the dense mitochondrial density and unique expression of uncoupling protein-1 (UCP1). The accumulation of adipocytes in bone marrow has also been observed during obesity and aging at the expense of osteogenic lineages, leading to the impairment of hematopoietic and bone regeneration [131].

TGF-β signaling has been shown to play an important role in the adipocyte differentiation of adipose stromal cells (ASCs) and bone-marrow-derived MSCs [130]. In general, the TGF-β/activin-Smad2/3 pathway promotes the proliferation of preadipocytes while inhibiting their differentiation into adipocytes. Smad3 physically interacts with C/EBP transcription factors and represses their transcriptional activity to induce PPARγ, the master regulator of adipogenesis [132]. Conversely, the BMP-Smad1/5/8 pathway apparently stimulates both proliferation and adipocyte differentiation. In addition, the TGF-β-Smad3 pathway has been shown to be important for the differentiation of brown adipocytes, the balance of WAT/BAT transition, and the modulation of obesity in several mouse studies. The genetic depletion of Smad3 and systemic treatment with anti-TGF-β antibody has been shown to protect mice from diet-induced obesity, insulin resistance, and hepatic steatosis [133,134]. These effects are associated with the Smad3-mediated suppression of PGC-1α, a transcriptional activator of mitochondrial biogenesis and UCP1 expression, and white-fat-to-brown-fat transition caused by Smad3 loss [133]. Furthermore, TGF-β has pro-diabetic roles in the CNS, especially the hypothalamus, which modulates peripheral glucose metabolism. The hypothalamic production of TGF-β becomes excessive under high-fat diet conditions, resulting in hypothalamic inflammation, hyperglycemia, and glucose intolerance [135]. An excessive amount TGF-β induces a hypothalamic RNA stress response, leading to the accelerated mRNA decay of IκBα, an inhibitor of NF-κB [135]. Thus, TGF-β signaling exacerbates obesity and diabetes via both peripheral and central actions. The peripheral and central effects of BMP signaling on adipogenesis and glucose metabolism have also been suggested [130]. The TGF-β pathway is also thought to mediate the adipose tissue fibrosis observed in obesity.

Similar alterations in TGF-β signaling appear to occur in both obesity and aging. The increase in plasma TGF-β1 levels associated with aging has also been observed in human obesity, correlating with body mass index; the expression levels of TGF-β ligands in adipose tissue also increase in obesity [131,136]. This observation may be associated with the altered differentiation potentials of MSCs such as increased adipogenic differentiation, impaired osteogenic differentiation, and fibrosis phenotype [137]. In addition, excessive TGF-β production in the hypothalamus has been observed under both obesity and aging conditions [135]. Thus, TGF-β signaling may share pathogenic roles in energy and glucose homeostasis during obesity and aging.

## 9. Conclusions

In this review, the relationships between TGF-β signaling and cellular senescence, stem cell aging, and aging-related pathology were summarized. Though the effects of TGF-β signaling are highly dependent on cell type and cellular context, many branches of growth inhibitory mechanisms are considered to promote senescent phenotypes (Figure 1). Based on many results regarding aging-related pathology (Figure 2), the effects of TGF-β signaling can be generalized into two aspects: (1) The impairment of TGF-β signaling in certain cell types, exemplified by a loss of neuroprotective activities in AD and a loss of TGF-β-mediated growth inhibition in cancer, and (2) the chronic elevation of TGF-β signaling linked to tissue fibrosis, chronic inflammation, and decreased regeneration capacity, as well as metabolic malfunction observed in AD, muscle atrophy, obesity, and other diseases. In addition, changes in context dependency may be important during aging. In studies on HSCs, the alteration of Smad2/3 partners, including Tif1γ, during aging has been suggested [79]. Furthermore, in studies on osteoarthritis, an age-related shift in the ALK1/ALK5 ratio altered TGF-β downstream signals by favoring Smad1/5/8 over Smad2/3 [87].

In recent years, numerous studies have revealed the various mechanisms of context-dependent effects of TGF-β signaling [138]. Nevertheless, the relationship between TGF-β signaling and aging remains unclear in many pathological conditions, and aging-dependent changes are an important issue warranting further investigation, which could provide a basis for the better modulation of TGF-β signaling in therapeutic approaches.

## Abbreviations

4-HNE	4-hydroxynonenal
ALK	Activin receptor-like kinase
ASC	Adipose stromal cell
AD	Alzheimer’s disease
ALS	Amyotrophic lateral sclerosis
Aβ	β-amyloid peptide
BMP	Bone morphogenetic protein
BAT	Brown adipose tissue
KLS	c-Kit^+^/Lin^−^/Sca-1^+^
CNS	Central nervous system
CSF	Cerebrospinal fluid
co-Smad	Common-partner Smad
DPMSC	Double positive mesenchymal stem cell
ECM	Extracellular matrix
GDF	Growth differentiation factor
HSC	Hematopoietic stem cell
IPF	Idiopathic pulmonary fibrosis
I-Smad	Inhibitory Smad
ITGB3	Integrin β3
MSC	Mesenchymal stem cell
MHC	Myosin heavy chain
PAI-1	Plasminogen activator inhibitor-1
ROS	Reactive oxygen species
R-Smad	Receptor-regulated Smad
Rb	Retinoblastoma
SASP	Senescence-associated secretory phenotype
SA-β-gal	Senescence-associated β-galactosidase
SIRT6	Sirtuin 6
TERT	Telomerase reverse transcriptase
TβRI	TGF-β type I receptor
TβRII	TGF-β type II receptor
Tif1γ	Transcriptional intermediary factor 1 γ
TGF-β	Transforming growth factor-β
UCP1	Uncoupling protein-1
UPR	Unfolded protein response
WAT	White adipose tissue
